# Technology to support aging in place: key messages for policymakers and funders

**DOI:** 10.3389/fpsyg.2023.1287486

**Published:** 2023-11-16

**Authors:** Courtney Genge, Heather McNeil, Patricia Debergue, Shannon Freeman

**Affiliations:** ^1^National Research Council Canada, Aging in Place Challenge Program, Ottawa, ON, Canada; ^2^School of Nursing, University of Northern British Columbia, Prince George, BC, Canada; ^3^Centre for Technology Adoption for Aging in the North, Prince George, BC, Canada

**Keywords:** older adults, health policy, implementation science, AgeTech, aging in place, gerontechnology, Canada

## Abstract

AgeTech, a subset of the health technology industry, uses technology to support healthy aging, and support care partners and health professionals to improve quality of life for aging adults. By enhancing and adapting alternative care approaches through emerging technologies, it is possible to enable and extend the ability for older adults to safely age in place within their own homes, improve care experiences, and/or decrease long-term care costs/needs. With the rapid development and proliferation of AgeTech into the consumer market, it is paramount for policymakers and funders to ensure that AgeTech solutions can be leveraged to support older adults to age well in place. This paper highlights five key messages for policymakers and funders drawing on experiences from Canada. First, it is essential to embrace a life course perspective on aging, recognizing the heterogeneity of older adults who experience diverse and evolving needs. AgeTech should adapt as needs and capacities evolve. Second, AgeTech should solve a real problem. Technology must be well aligned to the needs and preferences of older adults to be impactful. Third, health related AgeTech should empower, enhance, or support existing health care services, while recognizing the value of human interactions. In-person interactions can provide meaningful connection and important health data which should be enhanced not replaced. Fourth, the establishment and ongoing fostering of authentic partnerships to inform, co-create and co-design AgeTech solutions is key to developing successful products. Finally, policymakers and funders have an important role to play in enabling accelerated design, development and testing to meet current and future needs.

## 1 Background

Many countries, including Japan, Germany, and Italy are already considered “super-aged” with over 25% of their populations over 65. Many other nations, including the United States, Korea, Sweden, New Zealand, and Australia, are expected to reach this status by 2030 (United Nations, 2019). Similarly, the population of Canada is going through significant demographic shifts, with older adults (65+) becoming the fastest-growing segment and projected to represent 25% of the country’s population by 2050 (FP Analytics, 2018). While population aging represents accomplishment and positive opportunities for society, as the demographic landscape changes, there is a pressing need to adapt healthcare, social, and economic systems to support older adults and their caregivers effectively and ensure financial sustainability. Indeed, this shift represents such a significance, that the United Nations (UN) declared the years 2021–2030 as the Decade of Healthy Aging, to improve the lives of older people globally (The World Health Organization, 2018).

Older adults in Canada, and around the world, have articulated a strong preference to remain in their homes and communities as they age and the Covid-19 pandemic further strengthened this preference (Peek et al., 2014; Garner et al., 2018; Huyer et al., 2020; National Seniors Strategy, 2020). Aging in place is a multi-faceted, complex concept and is related to the UN Decade of Healthy Aging conceptualization. The core concepts of the World Health Organization’s (WHO) definition of Healthy Aging are functional ability, intrinsic capacity, and environmental characteristics. All of these need to be addressed in order to provide choices to older adults to age in homes and communities of their choice (The World Health Organization International, 2020). Further, the UN sustainable development goals (SDG) include the importance of supporting health and well-being (SDG 3) at the same time as ensuring Gender Equity (SDG 4), Reduced Inequities (SDG 10), Industry, Innovation, and Infrastructure (SDG 9) and Decent work and economic growth (SDG 5). These goals are critical to foster and sustain an AgeTech sector to support older adults to age in place (Antonucci, 2021).

### 1.1 Aging in place in Canada- challenges and opportunities

#### 1.1.1 Functional ability and intrinsic capacity are key considerations affecting Canadians ability to age in place in later life

Currently, older adults constitute 17% of Canada’s population but account for 47% of total healthcare costs (Canadian Institute for Health information, 2019). Aging is associated with the accumulation of cellular and molecular damage within the body which can manifest as frailty, cognitive impairment and/or chronic health conditions (The World Health Organization, 2022). This increases challenges that threaten older adults’ independence and subsequently their ability to age in place. Some challenges include medication management, injury, poorly controlled chronic conditions, frailty and cognitive impairment.

##### 1.1.1.1 Medication management

Many older adults are more likely to experience polypharmacy- defined as taking between 2–7 medications daily- which has been associated with increased risks of medication errors and adverse drug events (Shah and Hajjar, 2012). Research shows that 13% of people taking 2 or more medications will experience adverse events compared to 58% for those taking 5 or more and 82% for those taking 7 or more medications (Tsilimingras et al., 2003). Although medications are effective in combating diseases, their full benefits are often not realized because approximately half of patients do not take their medications as prescribed (Canadian Society of Hospital Pharmacists, n.d.). Medication non-adherence results in additional physician visits, extra laboratory tests, additional drug therapy, hospital emergency room visits, hospital admissions and readmissions, and short-term disability insurance payments.

##### 1.1.1.2 Injury

Injuries from falls, accidents, and motor vehicle collisions are also a major risk for older adults in Canada. More than 25% of older adults report at least one fall in the previous 12 months and falling once doubles the chances of having further falls (Pearson et al., 2014). Hospitalizations due to falls account for approximately 85% of injury-related hospitalisations for older adults in Canada annually (Tsilimingras et al., 2003)^.^ Over one third of those who are hospitalised for a fall are discharged to long-term care (Government of Canada Publications, n.d.).

##### 1.1.1.3 Poorly controlled chronic conditions

Many chronic conditions are ambulatory sensitive meaning they can be effectively managed in the home or community setting if there is an appropriate care plan, good health literacy and access to support. A 2011 report from the Canadian Institute for Health Information (CIHI) found that 76% of older adults reported having one or more of eleven chronic conditions, and being over the age of 60 was a significant risk factor for hospitalization with ambulatory sensitive conditions (Sanmartin et al., 2011). Hospitalization is associated with a number of preventable harms including deconditioning, delirium, and adverse events which can act as ‘sentinel events’ requiring transition to nursing home care (McAvay et al., 2006; Basic and Hartwell, 2015).

##### 1.1.1.4 Frailty

Frailty is a medical condition of reduced function and health in older individuals. The risk of becoming frail increases with age, but frailty is distinct from normal aging. Approximately 1.5 million Canadians are living with frailty and they are over-represented at all levels of the healthcare system (Canadian Frailty Network, n.d.). Older adults living with frailty are at higher risk of major deterioration and health decline following minor illnesses and are more likely to be hospitalised, need long-term care, or die (Nuernberger et al., 2018).

##### 1.1.1.5 Cognitive impairment

Approximately 500,000 older Canadians are living with some form of dementia (Chambers et al., 2016). People with dementia have twice as many emergency department visits and hospitalisations compared to peers, and every year approximately 25% of the population with dementia either visit the emergency department or are hospitalised (Godard-Sebillotte et al., 2021). A 2020 study by Huyer et al. found that a diagnosis of dementia was strongly associated with admission to a nursing home prior to death (Huyer et al., 2020).

These issues are all further amplified by shortages of health human resources (doctors, nurses, personal support workers, and social workers) who would typically support older adults to mitigate these risks.

#### 1.1.2 Environmental context is a key consideration also impacting older adults ability to age in place

The health related functional and intrinsic characteristics that older Canadians face are often compounded by social, financial, and infrastructural barriers that directly impact older adults’ abilities to age in place. At the systems level, one major challenge with the current healthcare system in Canada is that it was designed when the average age and life expectancy of the Canadian population was younger. Funding is commonly reactionarily allocated and focused on acute care with limited and often fragmented underfunding in primary and community care. The 2020 National Seniors Strategy for Canada and the Canadian Medical Association both state that the needs of older adults living in the community are inadequately met (National Seniors Strategy, 2020; Canadian Medical Association, 2021) increasing the risk of potentially avoidable transitions in care location and an increasing burden and demands of family caregivers. Recent Canadian studies suggest that up to 22% of older adults who had recently transitioned into nursing home care could have stayed at home had appropriate support been in place (Nuernberger et al., 2018; Canadian Institute for Health Information, 2020). However, across Canada the demand for home care services and support greatly exceeds the supply available (Home care Ontario, 2018). Furthermore, the regular turnover of staff and scheduling issues mean there are concerns about continuity of care and issues with patient safety when multiple providers are involved but not communicating effectively (Sanmartin et al., 2011).

A key social consideration impacting aging in place is family caregiver distress. As the number of older adults who need care and support increases, so do the number of family caregivers who provide unpaid care. In Canada, as in other countries, urbanization and increased mobility are impacting younger demographics, as they move from rural areas to towns and cities for better economic and social opportunities (Dandy and Bollman, 2009)^.^ This trend has resulted in rural and suburban areas aging faster than major urban centres, leading to a rising number of adult child caregivers living farther away from their aging parents (Canadian Institute for Health information, 2019). Research indicates that there are approximately 8 million Canadians who are currently caring for or supporting a family member. Almost half (47%) are caring for a parent or in-law and around 13% are caring for a spouse or partner (Statistics Canada, 2018). While caregiving can be a meaningful and rewarding activity, it can also result in emotional, financial, mental and physical stresses that place immense burden on the family caregiver. Family caregivers may experience stress from both objective burden - defined as tasks of care or physical requirements - and subjective burden - defined as the emotional or mental impact of caregiving (Montgomery et al., 1985). Presence of a family caregiver is typically protective against nursing home admission in the short term (Boaz and Muller, 1994), but distress, burden and burnout are strong indicators and predictors of a transition in care (Nuernberger et al., 2018). Recent analysis from the Canadian Longitudinal Study on Aging demonstrated that spousal caregivers were spending an average of 32 h per week, and adult child caregivers are spending an average of 20 h per week (Li et al., 2021). In Canada, caregiving responsibilities are estimated to result in the loss of 18 million work days per year, with a resulting cost of lost productivity estimated at $1.3billion. This illustrates the scale of the burden family caregivers are currently taking on.

In addition to social support, infrastructural support is necessary for aging in place. One such facilitator is the ability to move around one’s community. Older people typically cease driving approximately 10 years before they die and become dependent on others or mass transit to move about their communities (Turcotte, 2012). Mass transportation, including buses, trains, and airplanes, still have barriers that prevent fulsome use by many older adults (Patterson et al., 2019). Additionally, older adults can be particularly vulnerable to financial pressures. An increasing number of older adults fall into the category of “low income,” with the rate rising from 12% in 2005 to 14.3% in 2015 despite national poverty rates remaining stable (Waddell et al., 2018). This has translated into a growing number of older adults living in shelters or reporting being homeless or vulnerably housed (Reynolds et al., 2016). Older adults are frequent targets of cybercrime and financial abuse in Canada, resulting in an estimated annual cost of $650 million (Crane, 2019). Given these challenges and barriers to aging in place, there is growing interest in the potential for technologies to play a key role in supporting older adults and preventing transitions in care (Duan-Porter et al., 2020). The perception of older adults as “technophobic” is increasingly out of date as evidence gathered during the Covid-19 pandemic indicated that over 75 percent of older adults are confident in engaging with technologies (Freeman et al., 2022). However, adoption remains a challenge due to the social, infrastructural and financial barriers previously mentioned. Additionally, lack in understanding the needs, values, and preferences of older adults regarding current and future technologies result overall in less than 25% of older adults who are actively using technologies to support their health and wellbeing (Astell et al., 2020).

### 1.2 Agetech can be leveraged to support aging in place

Providing choices to older Canadians, their care partners, and health and social care systems is essential to meet the unique social, fiscal, and medical challenges associated with the needs of our rapidly aging population (Blackman et al., 2016). Technologies that are designed to improve the lives of older adults is one promising way to increase choice and support for older Canadians. The term to describe these innovative solutions is AgeTech, which refers to hardware or software solutions that aredesigned explicitly for or with the potential to provide benefit to older adults and their caregivers. This includes a range of innovations supporting aging in place, healthy aging, staying connected, and more. Given the complexity of aging and challenges to support aging in place, AgeTech includes a diverse portfolio of innovation from digital health, assistive technologies, Internet of things (IoT), medical devices/diagnostics, robotics, wearables and other sensor-based technologies. AgeTech includes digital technologies, digitally enabled technologies, and hardware solutions that support older adults to age in place. AgeTech is also referred to as GeronTech, ElderTech and SilverTech (Etkin, 2022). Assistive technologies- such as walkers, hearing aids, corrective lenses- are a subset of AgeTech, but older adults and their caregivers are now able to access a wider range of tech-enabled or enhanced approaches and technologies to support a holistic approach to healthy, active, socially connected aging. AgeTech tools and devices can help older adults to age in place by preventing transitions in care through improved health and wellbeing, enabling people to live well with advances in frailty and ill health, and/ or creating age-friendly communities and social structures. By enhancing and adapting alternative care approaches through emerging AgeTech, it may be possible to enable and extend the ability for older adults to safely age in place within their own homes, reduce and/or delay need for long term care facility supports, and/or decrease home care costs/needs (Freeman et al., 2023).

Information and Communications Technologies (ICT), defined as a diverse set of technological tools and resources used to transmit, store, create, share or exchange information (Zuppo, 2012), play an integral role in the daily life of most people (Freeman et al., 2020). The vast majority of newly developed or augmented technologies are ICT-based. Other types of technology specifically designed to support aging in place, such as emergency help systems, remote vital signs monitoring, and fall detection systems, are commonly referred to as smart home technology (Balta-Ozkan et al., 2013).

The ability to use ICTs, smart home technologies, and other digital tools is referred to as digital literacy (Gilster and Glister, 1997). Also adapted to support aging in place is the concept of electronic health or eHealth. WHO defines eHealth as the cost-effective and secure use of ICT in support of health and health-related fields. ICT has been demonstrated to reduce health system costs, while simultaneously improving care experiences for older adults in society (Mantovani and Turnheim, 2016). The Covid-19 pandemic was an accelerator for the proliferation of AgeTech for accessing health services, with the rise of virtual care, a subset of eHealth, and expanded investments in digital infrastructure across Canada (Sixsmith et al., 2022). However, there are still people who remain unable to access the necessary internet and digital resources to support reliable and ongoing access. These continued barriers in access to virtual care and digital infrastructure has further reinforced inequities and exacerbated what is commonly referred to as the digital divide (Fang et al., 2019; Freeman et al., 2022). To achieve equitable access and desired impact it is essential to ensure thoughtful technology design considerations, accounting for current digital literacy gaps as well as diverse levels of access to technology infrastructure, e.g., internet and cellular services (Health Canada, 2021). By leveraging these rapid technological advancements, AgeTech could revolutionize the aging experience as part of a systemic approach to supporting aging populations, by empowering and enabling older adults and their caregivers.

It is critical that AgeTech industries establish ample infrastructure to promote inclusive and sustainable industrialization as well as foster an innovation ecosystem which recognizes the changing needs and abilities of individuals as they age. While change and adaptation of AgeTech are necessary and inevitable, organizations and systems leaders must embrace these transformations and remain nimble and flexible to respond to expected and unforeseen changes. In Canada, the National Research Council of Canada (NRC) launched the Aging in Place Challenge Program in 2021 with a seven-year mandate and an overarching goal of developing technologies and innovations to support an increase in the number of older adults who remain in homes or communities of their choice by 2031. The program is collaborating with older adults and family caregivers as well as partners in academia, industry, and government toward enabling advancements in AgeTech in Canada. At a more regional level, the Centre for Technology Adoption for Aging in the North (CTAAN) supports aging in northern and rural communities by making technologies more available to older adults, caregivers, and the healthcare systems that support them (www.ctaan.ca). CTAAN is a collaboration between the University of Northern British Columbia (UNBC), Northern Health, and AGE-WELL, Canada’s National Technology and Aging Network.

Through an iterative approach, CTAAN researchers, NRC scientists and their partners including an advisory panel of older adults and caregivers have been leveraging learnings from their collaboration to equip AgeTech industry leaders, community champions, and health decision-makers with practical skills and guidance. In doing so, they aim to enhance AgeTech to be more accessible, inclusive, responsive, and sustainable over time. This paper highlights five learnings from our experience to date, and then presents implications for policymakers and funders when considering the role of AgeTech to facilitate aging in place. The lessons shared in this paper emerge from preliminary research findings, process evaluations, and feedback from partners including scientists, granting agencies, older adults and community care organizations. These experiences stem from Canadian context, but the messages may be generalizable across diverse portfolios and geographies.

## 2 Key messages

### 2.1 Key message one: It is essential to embrace a life course informed perspective on aging, as older adults are heterogeneous and experience diverse needs that evolve over time

Taken individually and at a specific point in time, technologies may be able to effectively solve a problem however the sustainability of a proposed solution and its impact may be reduced if designers and policymakers fail to embrace a life course informed perspective on aging. The term “older adult” serves as an umbrella phrase encompassing individuals aged 65 and above, yet it is imperative to acknowledge the remarkable diversity within this cohort. Age is not only limited to a chronological number. Indeed, aging among individuals can differ greatly by biological aging, physical aging, and social aging processes. This may lead to great diversity experienced across chronological ages.

Older adults have a mosaic of profiles which intersect with factors such as age, gender, race, disability status, and geographical location. A “life course perspective on aging” is useful to understand this diversity. The life course perspective on aging is a well-recognized theoretical framework that highlights the impact of individual experiences and unique trajectories throughout life (Mayer, 2009), as well as the social forces that influence the experience of aging. It is now widely adopted across sectors, including in public health where it provides a foundation for policy focusing on improving health and health equity (Mayer, 2009). The life course perspective encourages a holistic approach to understanding how past experiences and capabilities influence experiences of aging taking into account both individual chronological age as well as cohort membership (Dannefer and Kelley-Moore, 2009). While individual characteristics affect experiences of aging, factors impacting diversity across the life course are often also interpersonal or socially determined in nature (Settersten, 2017).

Given the objectives of AgeTech it is crucial to be attuned to the diversity that accompanies the aging experience to ensure that designs account for a multitude of preferences and capabilities. The profile of those easiest to reach for health research and engagement is typically Caucasian, urban dwelling, well-educated, individuals who possess high levels of technological literacy (Bonevski et al., 2014). However, failing to engage with a sample representative of a broader population may result in research data that is non-generalizable, and potentially not applicable to those groups who have the highest burden of disease or most need (Sydor, 2013). To create technologies that meet the needs of older adults and their caregivers, diverse stakeholders must be consulted and engaged throughout the innovation process. Involvement of these older adults and caregivers in health and aging innovation can result in new technologies and processes that are more likely to meet their needs and preferences (McNeil et al., 2022). In addition, approaches to knowledge generation and implementation science that involve the collaboration of multiple academics across scientific disciplines and other experiential non-academics across sectors (e.g., industry, policymakers, health professionals) has been recognized as best practice in this area (Sixsmith et al., 2021).

Another key consideration for designing technologies for older adults, is the potential for the needs and capabilities of the end user to evolve with time. There is a clear link between aging and the development of certain health conditions such as frailty, dementias, musculoskeletal conditions, and sensory impairments. Good examples of this phenomena can be found in e-readers that offer both traditional reading and audiobook options to enable older adults to engage with literature in a variety of formats. The duality of modes in e-readers allows the older adult to continue using the technology they are familiar with even if they develop a cognitive or sensory impairment that makes traditional reading difficult. Another good example can be found in smartphones that have a variety of accessibility options such as modifiable text size, voice to text communications, and variable levels of access security that can be enabled or disabled based on user preference and capability. Embedding these evolutionary capacities into technology is aligned with the principles of user centric design which theorises that the technology should be built around, and adapt to the user and not the other way around, ensuring high degrees of usability (International Organization for Standardization, 1999).

Failing to recognize and embrace the complexity of aging in the process of AgeTech design and evaluation can lead to technologies that fail to effectively address the multi-causal pathways toward impact. This in turn hinders innovation adoption by older adults and their caregivers, limiting sustained utilization. One common critique of technology acceptance models is that they fail to account for evolutionary capacity and adapting functional ability (Peek et al., 2014). The most effective AgeTech is designed with evolving capacity and capability in mind to enable the older adult to continue to utilize and benefit from the technology over time.

Embracing a life course perspective is essential to ensuring research on aging in place is reflective of the diversity of experiences that older adults have over time. There is an opportunity to design more equitable policies and funding opportunities when embedding elements of life course theory. An example of good practice in this area from Canada is the Canadian Institute of Health Research’s (CIHR) Strategy for Patient-Oriented Research (SPOR).^1^ With a mandate to catalyze patient-oriented research, the ultimate goal of SPOR is to improve health outcomes through evidence-informed care. To do this, SPOR partners with various levels of government, researchers, health providers, patients, and other key parties to create hubs of expertise and fund research in areas of importance to patients themselves. In practice, the SPOR network engages experts by lived experience across the country across various levels such as setting the research agenda, advising on initiatives and participating in health related research.

### 2.2 Key message two: Agetech should solve a real problem

AgeTech must be aligned to the actual wants and needs of older adults and caregivers for innovation to facilitate aging in place. It is important that technology solutions are well aligned to the day to day challenges older adult and caregiver end users face. Ensuring that the problems they experience are being appropriately addressed is key to successful AgeTech development and deployment.

Technology development is rarely undertaken by older adults themselves, and thus the onus is on developers and researchers to ensure they have a solid understanding of the experiences and needs of older adults. In 2019 a review, Wang et al., found that a key barrier to technology adoption is “top-down” design process that rely on technologists preconceptions of the needs of older adults with little consideration of user perspectives and preferences or their real-world constraints (Wang et al., 2019). The growing complexity and diversity of healthcare management for older adults requires thoughtful approaches to identifying what challenges are appropriate for a technology enhanced/supported solution. Failing to design for real problems that older adults are facing risks designing technologies that add layers of complication and confusion for older adults without having a substantive impact on their wellbeing or ability to age in place.

Several factors that influence adoption of technology for aging in place include: (1) user concerns (e.g., high cost, privacy implications and usability factors); (2) expected benefits (e.g., increased safety and perceived usefulness); (3) user needs (e.g., perceived need and subjective health status); (4) available alternatives (e.g., help by family or spouse); (5) social influence (e.g., influence of family, friends and professional caregivers); and (6) user characteristics (e.g., desire to age in place) (Peek et al., 2014; Macedo, 2017). It is essential to ensure there is appropriate alignment of the needs, preferences and intended uses of the technology with the end users context otherwise there may be an elevated risk that the technology will be used sub-optimally or not used at all (Scherer, 2017). Barriers such as the concern for privacy and issues of trust (Canadian Institute for Health Information, 2020) must be addressed in order for technology to be perceived as acceptable. This illustrates the importance of the technology being well aligned with challenges or barriers older adults are facing in order for it to be perceived as useful and therefore acceptable. When adopting technologies for use, older adults balance between degree of privacy for the benefit of staying in their home (Jaschinski and Ben Allouch, 2019). Research has shown that older populations are very aware of privacy issues (Al-Shaqi et al., 2016; McNeill et al., 2017) and that privacy considerations are key factors in the adoption of assistive technologies. Privacy may be a larger issue for technologies designed for aging in place, particularly since older populations with health issues must learn to manage their personal health data (Kolkowska and Kajtazi, 2015). Older adults have also expressed preferences for what functions technology is used to support. For example, older adults report an interest in robotics that support instrumental activities of daily living (e.g., housekeeping, laundry, medication management) and enhanced activities of daily living (e.g., entertainment, hobbies, learning opportunities), but are hesitant to embrace robotics that support personal care activities (e.g., bathing, shaving etc.) (Smarr et al., 2012). This may be attributable to concerns about social isolation and loneliness if technology is perceived to replace in person interaction with professional and familial caregivers (Clayton and Astell, 2022).

Caregivers also commonly express openness to technologies that can support others to age in place, and intergenerational encouragement is identified as a key driver of adoption (Freeman et al., 2020). As many caregivers in the Canadian context are providing support from geographically dispersed locations, technology is uniquely positioned to enable long distance support. Technology is perceived by caregivers as an effective means of reducing caregiving burden as it can decrease reliance on in-person interactions. For example, automated medication management solutions can reduce the need for daily in person visits to ensure medication adherence. However, some caregivers fear that technology may add to their burden by making them more available and increasing the volume of monitoring and caregiving tasks they are responsible for Madara Marasinghe (2016). Ensuring technologies are solving for ‘pain points’ without adding complication or additional tasks for caregivers is key to ensuring the solution will be embraced.

It is essential for technology developers and researchers to conduct robust gap analysis and ensure technologies being proposed or designed are aligned with the needs and preferences of older adults and caregivers. Through its Calls for Innovation, the CAN Health Network^2^ is an example of encouraging this needs based way of working. The CAN Health Network supports joint-problem solving between companies and participating health systems in Canada. Innovation through CAN Health begins with health care system partners putting forward user-defined needs from their organizations for which they would be ready to procure a solution. Technology products are then identified to address these needs and research projects are co-created to ensure market value and system readiness of the technology are evaluated.

### 2.3 Key message three: Agetech should complement and support existing health care and social services and supports, not functionally replace human resources

It is essential to design AgeTech that complements, enhances, and supports the healthcare system. The demand for care in Canada far exceeds the resources and budget available, leading to many older adults struggling to access “best practice” care and support. There is a growing need for technologies that can support and complement the strained healthcare system without aiming to replace highly skilled human resources.

Older adults have clearly indicated their preference for shared decision making, circle of care approaches, and relationship centric approaches that include care partners (Elliott et al., 2016). These attributes are most effectively embedded in care approaches that enable continuity of care and ongoing relationships between practitioner and patient. This is particularly notable for those who receive ongoing support for activities of daily living (such as assistance with dressing, bathing, and personal care). Where technologies are leveraged to streamline care processes, augment care, improve outcomes or support better clinical decision making, the innovation is welcomed. For example, older adults were the highest users of virtual care during the Covid-19 pandemic as the users benefited most from avoiding in-person visits and the corresponding increased risk of serious infections (Bhatia et al., 2021). However, where technology is viewed as replacing a human resource, replacing in person interactions, or increasing workload, then there is increased hesitancy to embrace that technology. This may be attributable to perceptions that accepting technology may be a gateway to social isolation (Clayton and Astell, 2022).

Additionally, there is a risk that replacing an in-person interaction with a technology may result in missed diagnosis or poor care outcomes. Geriatric medicine is by nature complex, and distilling care down to narrowly defined data points provided by a technology without being able to access broader contextualization risks missing important data. Providers of geriatric care use multiple sources of information, including observation, clinical measurements, and patient self report as a barometer for overall health and functionality. These multiple convergent sources of information allow the high skilled practitioner to tease out nuances and important details. Geriatric medicine often focuses on “ability to live well with chronic conditions” which differs from many other branches of medical care that tend to focus on restorative or curative functionality. Geriatric practitioners frequently focus on “the 5 M’s of geriatrics” – mind, mobility, medications, multi-complexity, and ‘matters most’. Matters most is a key element of geriatric medicine that focuses on the personal preferences and values of the individual to support enhanced care planning, defining goals of care and ultimately defining preferred outcomes (Health in Aging, 2019). This is achieved meaningfully through cultivation of a relationship between patient and care provider.

Nevertheless, there are many opportunities to integrate technology into care for older adults that can improve, enhance and support practitioners without replacing their clinical judgment. Technologies that provide data from the home context may be able to more accurately assess function and risk as they provide data from people’s typical living circumstances vs. lab or hospital based data (Wu et al., 2023). Assessment of capability in hospital setting versus the home setting is a known risk for transition to nursing home care (Nuernberger et al., 2018) which may be attributable to routine underestimation of ability by hospital staff (Bender and Holyoke, 2018). Consumer availability of smart devices and wearables has led to an abundance of potentially relevant clinical data being collected. The primary question is how to integrate that data from smart devices and wearables into clinical practice in a way that is accessible, acceptable, and helpful. Technology may also have a role in reducing time, effort, or human resources for administrative tasks that are currently being done by skilled health resources. It is not sustainable for caregivers (professional or familial) to continue to be asked to do more with less. Technologies should aim for “zero- effort” and output data that are easy to interpret and integrate into routine activities. Interoperability and data sharing will play a key role in enabling data to be meaningfully shared and actioned.

Funders can support the development of technologies that assist health systems by requiring partnership with frontline care providers in codesign. Providing funding for use case development and validation could also ensure alignment between development objectives and health system needs. The *Fonds de soutien à l’innovation en santé et services sociaux* set up as part of the Quebec Life Sciences Strategy of the Quebec Ministry of Health in collaboration with the Ministry of Economy and Innovation, is a good example of coordination of efforts toward accelerating adoption of relevant innovations [Fonds de soutien à l’innovation en santé et en services sociaux | Ministère de l'Économie, de l'Innovation et de l'Énergie (gouv.qc.ca)]. This initiative aims to support the *Bureau de l’Innovation* mandate to integrate innovation. It is intended to provide financial support for projects that test the validity and usefulness of innovations in a real health care and service environment.

### 2.4 Key message four: Partnership with the right collaborators is key to AgeTech success

While end-user engagement is recognized as “best practice” in design, integration of these principles in AgeTech innovation has been slow to become mainstream. Designing from a position of partnership means restructuring the relationship between designer and end-user in a way that recognizes older adult end-users as experts through lived experiences, and designers as experts through technical or scientific knowledge (Manafò et al., 2018). There are several benefits to codesign which include (i) creative idea generation through the sharing of knowledge, (ii) increased speed of adoption of interventions due to local ownership, (iii) development of interventions which are more inclusive and accurately reflect user experience, (iv) increased user satisfaction with services, and (v) lower costs for the organizations implementing the interventions (Steen et al., 2011).

Emphasis on end user involvement, especially when engaging older adults and their care partners, is constantly challenged by the scarcity of resources available to facilitate substantive involvement and the limited capacity of staff who are often juggling multiple responsibilities. All too often, engagement work is treated as “off the side of the desk” or allocated to a “champion” who takes responsibility for embedding the approach across multiple projects. End user engagement takes time and skills and must be embedded within standard procedure otherwise it risks becoming a tokenistic gesture.

When undertaking codesign and engagement it is important to include end users with a diversity of perspectives and experiences. It is essential to consider the heterogeneity of the older adult population and plan engagement accordingly. Inclusive design principles stress the importance of including marginalized representation in the sample as the needs of individuals at the margins are typically more diverse. Considering those needs reinforces the inclusive character of the technology solution (Inclusive Design Research Centre. OCAD University, 2023). The responsibility for creating an environment that is conducive to engagement lies with researchers and developers. These stakeholders can create foundations for successful engagement by educating themselves on relevant equity, diversity and inclusion considerations as well as integration of gender-based analysis.

Co-production is a long-term process, and change will happen at the speed of trust between the partners. To speed up the process, it is helpful to partner directly with organizations that already have trusting relationships with end users (Bonevski et al., 2014). This may include SPOR programs, advocacy networks, and community based organizations. Partnering with organizations or individuals who have existing relationships enables developers to benefit from the foundations of trust and authenticity while still working to the necessary timelines for agile technology development.

An example of this partnership in action is illustrated by ‘AgeTech Discussions which Explore User Perspectives on Technology’-referred to as ADEPT workshops. These workshops are a key service CTAAN provides to introduce AgeTech to potential users. ADEPT workshops showcase an emerging AgeTech to stakeholders in northern and rural areas, describing the applicability, usability, and feasibility of a featured AgeTech from end users’ perspectives. Through workshops, end users participate in facilitated discussions and provide important insights and recommendations to inform design and adjustments of featured AgeTech. This process provides technology developers and companies with evidence that helps form the next steps to scale their products and services to northern and rural areas. At its core, the ADEPT reports generate new evidence to inform AgeTech leaders of the utility, feasibility, and perspectives from Canadian health systems leaders, healthcare providers, care partners, and older adults. CTAAN has spent years developing trusting relationships with collaborators in the community which has created a shared language between diverse individuals and the facilitators at CTAAN.

Policymakers and funders can support meaningful engagement and partnership development by encouraging research and AgeTech development teams to include staff dedicated to the role of community engagement. Furthermore, policymakers and funders can support longer research fellowships, and provide living wages for highly qualified personnel to allow trainee’s time to build meaningful relationships with community partners. This may avoid academic precarity or cost of living concerns impacting their research productivity and career progression.

### 2.5 Key message five: Agetech design, development and testing needs to be faster and more flexible to meet current needs

Digitalisation offers opportunities to address current health and care system challenges. There is agreement from stakeholders across the Canadian AgeTech ecosystem that technology design, development and testing need to move faster and be more flexible to meet the existing needs of today (Desveaux et al., 2017). When tackling complex problems, a pragmatic approach is best suited. There is a need to challenge the existing hierarchy of research evidence that venerates clinical trials as the “gold standard (Greenhalgh and Russell, 2010). When it comes to evaluation there is a mismatch between the underlying philosophies of clinical trials and the pragmatism needed for AgeTech evaluation.

One reason AgeTech is not always well suited to clinical trial methodology is that the complexity of aging related illness, and concurrent complexity within the healthcare system cannot be replicated or randomized in an artificial lab setting. Complex problems demand complexity informed evaluations. In contract, clinical trial methodology requires the researcher to control for complexity and externalities. This is particularly relevant when assessing technologies for aging in place as the logical indicator of success is perceived to be avoidance of transition in level or location of care. However, the decisions about aging in place are multifactorial and generally involve multiple stakeholders. It can be extremely difficult to isolate the impact of a technology on that complex decision-making process. If appropriate, and contextually sensitive evaluation metrics are not selected, a novel technology may be deemed a “failure” or “non-impactful” when an individual using that technological intervention moves into a formal care setting. When focusing only on that outcome measure, the evaluation will not capture the actual impact or “successes” of the technology.

This challenge is compounded by the tension that is reported between researchers and technology developers when “ways of working” are perceived as incompatible. Adaptation and adjustment of research structures are needed to foster successful collaboration. Both of which require flexibility and transparency in timelines, process, and expectations. For example, in traditional research structures the process of gaining research ethics approval, especially if multiple academic and clinical boards are involved, can take several months. This timeline represents a delay which is unacceptable to many developers. However, with effective partnerships it is possible to create overarching research infrastructure. One such tool is an umbrella ethics approval that cover the research team and key research activities. Tools such as these allow for each new development opportunity to be treated as an amendment to the approved ethics application. This approach, which is used by CTAAN to quickly respond and provide ADEPT workshops, enables more rapid turnaround and progression of development research, while also ensuring the principles of ethical research are upheld.

Another useful tool is the minimum shared data set. This data set comprises metrics that matter most to each of those most affected parties in the development process. Having the core dataset agreed upon in advance will ensure that technology iteration and development can occur alongside continuing academic research. This approach again ensures that technology development is backed by core research principles while addressing the issue of incompatible timelines between academic research and industry. An additional benefit of the minimum shared data set also is the potential to support technology adoption into health systems. These systems have timelines that are often perceived as slow, are generally risk averse and rely heavily on evidence and data to inform decision making. Co-creating the minimum data set with health system partners or intended adopters at the outset of a design and development process ensures that the outputs of the project will be aligned to the key evaluation metrics the adopter will be assessing the final output against. This can avoid duplication of effort and delays in the innovation to adoption pipeline. Partnership with the right stakeholders is key to effective development of usable, accessible and helpful technologies.

It is well known and recognized that healthcare systems tend to slowly adopt new technologies because lives could be at stake and safety and security always come first. As a result, the digital health industry moves faster than what the systems are able to absorb, AI-based technology adoption is lagging and regulatory bodies have difficulties adapting with rapidly changing innovations. While individuals may desire access to digital healthcare it is not being used to its full potential in Canada because technology adoption is slow. Reported barriers include geographic variation in payment models, licensing, and regulation requirements across Canadian provinces and territories (Virtual Care Task Force, 2020).

Policymakers and funders can support agile technology development by providing funding opportunities that align with industry preferred approaches while simultaneously supporting longer term research projects that are complexity informed. Encouraging policymakers to facilitate industry opportunities that create shorter term, rapid funding cycles to allow researchers and developers to iterate will support refinement and technology progression. Ensuring research funders are concurrently facilitating longer research cycles to allow research teams to embrace longitudinal, complexity informed evaluation will help generate more meaningful and contextual impact evaluations.

A recent example of a funding opportunity from Canada that embedded these elements was CIHR’s eHealth Innovations Partnership Program (eHIPP) (Canadian Institutes of Health Research, 2017). This was a collaborative funding program designed to create healthcare innovation by funding projects with partnerships between Canadian technology companies, researchers, and health care system partners. The funded projects needed to articulate a methodology of co-development and a focus on integration plans for their innovative e-health solutions. The program supported pragmatic evaluation to ensure that the technologies would deliver real-world health care value.

There is a need to progress work on AgeTech standard based solutions that provide guidance on metrics and a shared framework for design and evaluation. Policymakers and funders can support the development and sharing of data to inform AgeTech evaluations. This approach will balance the need for academic rigour and agility by ensuring high impact metrics are prioritized to support technology integration. Funders should ensure that these are developed by multidisciplinary partners, including industry and experts by lived experience as it is recognized that together these partners can provide valuable insights.

A recent example of this is the NRC Aging in Place Program actively supporting the development of AgeTech standard based solutions for design and evaluation. Through a multidisciplinary project the objective is to co-create recommended AgeTech design and evaluation frameworks, guidelines, and best practices, to expedite the development, validation and dissemination of effective technologies that can address the needs of older adults in a safe and reliable way. The project will explore best practices, guidelines and standards for AgeTech that might be used by innovators and industry (i.e., technology developers and adopters) to expedite time to market and help guide the choices of older adults and their care partners to age more independently in the place of their choosing. This project will employ a multidisciplinary perspective and will include experts by lived experience to ensure that the outputs matter to users of AgeTech.

## 3 Discussion: Implications for policymakers and funders

Policymakers and funders are key to directing how AgeTech research and development evolve. They must work collaboratively to ensure organizational policies, structural mechanisms and application parameters support the good practices proposed here. Through thoughtful authentic codesign and deliberate structure of funding mechanisms, policymakers and funders can ensure good practices are embedded within future research. Below are some recommendations:

Ensure life course theory and complexity informed evaluations are embedded in AgeTech development. Policymakers and funders can play a key role in encouraging and enabling these perspectives though careful design considerations when structuring calls for applications. Experts by lived experience need to be engaged throughout the innovation process. This collaboration will allow for the challenging of assumptions and opportunities for building equity considerations specific to older adults. To encourage diverse life course perspectives, funders can require older adults to be part of a research team as project partners or as members of an advisory board. Funders can also include older adults as part of their review panels to ensure the perspective of end users in evaluating funding applications.

Prioritize policies that remove barriers and enhance participation of hidden, hard to reach, and seldom heard populations. For example, funders can include participant payment and reimbursement of expenses to remove financial barriers to participation. Policymakers and funders should also consider allowing payments for respite care to enable caregivers who have sole or primary responsibility to participate in research and design without the barrier of care expense or anxiety about leaving the care recipient alone. This is already considered ‘good practice’ in research, but is not embedded into many funding calls.

In recognition of the essential need to engage with diverse populations, policymakers and funders should ensure Equity, Diversity and Inclusion (EDI) and Gender-Based Analysis Plus (GBA Plus) plans are mandated components of granting opportunities. Training opportunities for highly qualified personnel, scientists, and staff involved in AgeTech development in EDI/GBA plus can support the creation of meaningful EDI/GBA plus plans that ensure the principles of recognizing and redistributing power, and including perspectives of diverse individuals throughout the innovation process.

AgeTech funding should support dedicated staff members to work on relationship building and engagement activities. To ensure co-design and collaboration are done in a fulsome way, it is essential to recognize that engagement and codesign are distinct skills. Funders should articulate that research teams have such dedicated personnel and include description of this role and experience in this work as part of the evaluation criteria. Policymakers can also ensure the terms of funding allow for “flow throughs” to community organizations to enable research teams to partner with community organizations that have existing relationships with those who have “often ignored perspectives.” Lastly, funders can encourage research teams to critically consider “who is missing” from the discussion and include plans for how to engage those participants in their funding applications. This action of requiring an appraisal of missing collaborators upfront can ensure funding goes to research teams that are able to effectively capture a life course informed perspective.

Embrace design approaches that allow teams to rapidly iterate and refine AgeTech. This may include rapid review and fundings cycles to allow teams to create use cases and prototypes and validate concepts with the community. Funding opportunities must be designed to enable teams to “fail fast” and “fail smart,” allowing them to learn from feedback and rapidly iterate. Rapid iteration will encourage better alignment between tech developers and end users by enabling early and frequent feedback during the development cycle without unduly hampering the commercialization process.

Favor methodologies and practices needed to study complex or “wicked” challenges. By supporting research that utilises methodologies that are pragmatic in nature and leverage multi-method evaluation, complexity can be properly considered. Aging in place is multi-faceted and frequently involves multiple decision makers. To fully explore the effect of technology on the ability to age in place, researchers must be able to interact longitudinally with multiple partners. When longitudinal studies are most appropriate, funders can embrace complexity informed theories of change as part of the funding call to ensure that scientists and those collaborating on a project are gathering the evidence needed to demonstrate impacts of AgeTech.

Integrate robust needs and gap analysis into funding application processes. It is insufficient to simply suggest or recommend inclusion of robust gap analysis; it must be structurally embedded within the funding mechanisms and directly tied to application evaluation criteria. Embedding these measures within application structure provides a mechanism to standardize evaluation and highlights the importance of gap analysis as part of project design. To keep up with the rapid pace of new and evolving AgeTech innovations entering the consumer market, these analyses must be conducted in a timely manner and tailored to address the information needs of the user and/or the consumer. Funders can encourage or require multidisciplinary teams to include healthcare professionals which helps ensure proposed innovations will complement, enhance and support the healthcare system.

Finally, move away from funding applications which merely showcase novelty of an innovation. Instead, require applicants to concretely demonstrate potential for tangible impact. For theoretical science, low Technology Readiness Levels (TRL) and developmental projects this may take the format of including named older adult and care partner experts by lived experience on the project team. Ensuring consideration of end users is embedded in the project design from the point of ideation provides assurances on real world needs. For higher TRL products or evaluations, funding applications should include a section for applicants to showcase previous engagement with end users and community groups. Previous engagement should highlight articulated potential use cases from the perspective of experts by lived experience. Embedding this content requirement into funding applications should catalyze behavior change as funding applicants are required to engage end users to access further funding.

## 4 Conclusion

Technology can be an effective means of supporting aging in place as it can provide rigour and objectivity to clinical decision making, supporting older adults and their caregivers and augmenting strained health human resources. However, there is a risk that technology without proper design and evaluation considerations may add layers of complication to an already complex system without actually creating the impact that is intended. The goal of AgeTech must be to improve outcomes by offering support, decreasing burden, and expanding access to resources, without causing undue confusion or stress for older adults and their caregivers. It is crucial to avoid placing excessive burden on caregivers, whether professional or familial. It is no longer the case that healthcare providers and care partners can be called upon to do more with less. Instead, sustained implementation of AgeTech must offer tangible benefits and solutions in terms of time savings or improved outcomes for caregivers. Technology needs to be an asset rather than a source of additional stress, and should remain accessible to all to avoid accentuating inequities.

Policymakers and funders must consider demographic shifts and proactively ensure the well-being and proper support of older adults and their caregivers in Canada. Technology has the potential to play a pivotal role in addressing challenges associated with aging in place. To do so it must be thoughtfully designed, developed, and implemented. AgeTech design and evaluation should be pragmatic. Designers, scientists, and policymakers should partner with older adults and their caregivers to better understand needs and implement impactful solutions. Real-world evaluation of AgeTech solutions should be conducted to quantify the investment and support required to foster sustained use including human, financial, education resources. With the rapid development and proliferation of AgeTech into the consumer market, it is paramount to cultivate an innovation ecosystem that ensures AgeTech solutions are co-created and evaluated in a good way in order to support older adults to age well in place.

## Author contributions

CG: Conceptualization, Project administration, Writing – original draft, Writing – review & editing, Methodology. HM: Conceptualization, Writing – original draft, Writing – review & editing, Validation. PD: Writing – review & editing, Supervision, Validation. SF: Conceptualization, Writing – original draft, Writing – review & editing, Formal analysis.

## Footnotes

^1^ https://cihr-irsc.gc.ca/e/51036.html

^2^ canhealthnetwork.ca
